# SPIRE – combining SGI-110 with cisplatin and gemcitabine chemotherapy for solid malignancies including bladder cancer: study protocol for a phase Ib/randomised IIa open label clinical trial

**DOI:** 10.1186/s13063-018-2586-7

**Published:** 2018-04-03

**Authors:** Simon Crabb, Sarah J. Danson, James W. F. Catto, Cathy McDowell, James N. Lowder, Joshua Caddy, Denise Dunkley, Jessica Rajaram, Deborah Ellis, Stephanie Hill, David Hathorn, Amy Whitehead, Mihalis Kalevras, Robert Huddart, Gareth Griffiths

**Affiliations:** 1 Southampton Experimental Cancer Medicine Centre, University of Southampton, Southampton, UK; 2 Academic Unit of Clinical Oncology, Weston Park Hospital, University of Sheffield, Sheffield, UK; 3 Academic Urology Unit, The Medical school, University of Sheffield, Sheffield, UK; 4 Cancer Research UK, London, UK; 5 Astex Pharmaceuticals, Inc., Pleasanton, CA USA; 6 Southampton Clinical Trials Unit, Centre for Cancer Immunology, University of Southampton, Southampton, UK; 7 The Institute of Cancer Research, Sutton, UK

**Keywords:** Urothelial bladder cancer, Guadecitabine, DNA methyltransferase inhibitor, Phase I/II, Randomised controlled trial, Cisplatin resistance

## Abstract

**Background:**

Urothelial bladder cancer (UBC) accounts for 10,000 new diagnoses and 5000 deaths annually in the UK (Cancer Research UK, http://www.cancerresearchuk.org/health-professional/cancer-statistics/statistics-by-cancer-type/bladder-cancer, Cancer Research UK, Accessed 26 Mar 2018). Cisplatin-based chemotherapy is standard of care therapy for UBC for both palliative first-line treatment of advanced/metastatic disease and radical neoadjuvant treatment of localised muscle invasive bladder cancer. However, cisplatin resistance remains a critical cause of treatment failure and a barrier to therapeutic advance in UBC. Based on supportive pre-clinical data, we hypothesised that DNA methyltransferase inhibition would circumvent cisplatin resistance in UBC and potentially other cancers.

**Methods:**

The addition of SGI-110 (guadecitabine, a DNA methyltransferase inhibitor) to conventional doublet therapy of gemcitabine and cisplatin (GC) is being tested within the phase Ib/IIa SPIRE clinical trial. SPIRE incorporates an initial, modified rolling six-dose escalation phase Ib design of up to 36 patients with advanced solid tumours followed by a 20-patient open-label randomised controlled dose expansion phase IIa component as neoadjuvant treatment for UBC. Patients are being recruited from UK secondary care sites. The dose escalation phase will determine a recommended phase II dose (RP2D, primary endpoint) of SGI-110, by subcutaneous injection, on days 1–5 for combination with GC at conventional doses (cisplatin 70 mg/m^2^, IV infusion, day 8; gemcitabine 1000 mg/m^2^, IV infusion, days 8 and 15) in every 21-day cycle. In the dose expansion phase, patients will be randomised 1:1 to GC with or without SGI-110 at the proposed RP2D. Secondary endpoints will include toxicity profiles, SGI-110 pharmacokinetics and pharmacodynamic biomarkers, and pathological complete response rates in the dose expansion phase. Analyses will not be powered for formal statistical comparisons and descriptive statistics will be used to describe rates of toxicity, efficacy and translational endpoints by treatment arm.

**Discussion:**

SPIRE will provide evidence for whether SGI-110 in combination with GC chemotherapy is safe and biologically effective prior to future phase II/III trials as a neoadjuvant therapy for UBC and potentially in other cancers treated with GC.

**Trial Registration:**

EudraCT Number: 2015–004062-29 (entered Dec 7, 2015)

ISRCTN registry number: 16332228 (registered on Feb 3, 2016)

**Electronic supplementary material:**

The online version of this article (10.1186/s13063-018-2586-7) contains supplementary material, which is available to authorized users.

## Background

Urothelial bladder cancer (UBC) accounts for 10,000 new diagnoses and 5000 deaths annually in the UK [1]. Cisplatin-based chemotherapy is a standard of care therapy for UBC in both the palliative first-line setting of advanced/metastatic disease and for radical neoadjuvant treatment of localised muscle invasive bladder cancer (MIBC) [2–4]. Globally, the most common regimen is either a doublet combination with gemcitabine (GC) or in combination with methotrexate, vinblastine and doxorubicin [4, 5]. Attempts to replace cisplatin as a component of chemotherapy regimens, for example, with carboplatin, have been unsuccessful in terms of reduced efficacy in UBC [5]. For metastatic disease, GC results in a median survival and time to progressive disease of approximately 14 and 7 months, respectively. Cisplatin-based neoadjuvant chemotherapy, prior to either radical cystectomy or radical radiotherapy for MIBC, adds an absolute survival advantage of 5–6% to overall cure rates, averaged across T stages at approximately 50% [2, 3, 6].

Cisplatin resistance remains a critical barrier to therapeutic advance in UBC. For example, in a key randomised trial comparing cisplatin-based regimens for advanced disease, 17% of patients had primary refractory disease and, by 3 years, only 13% were alive and free from progression [7]. Progression or relapse of UBC following cisplatin-based chemotherapy is associated with a dismal prognosis. For patients receiving second-line palliative chemotherapy or immunotherapy, after a prior platinum-based regimen, the median survival is consistently under 1 year with a median progression-free survival in the range of 2 to 5 months [5]. Recent data for second line use of palliative immunotherapy with the programmed cell death protein 1 inhibitor pembrolizumab has established a modest improvement in median overall survival of 10.3 months for the pembrolizumab group compared to 7.4 months in the chemotherapy arm [8]. To date, no biomarker-directed stratified treatment strategy has been established for use in UBC [5]. Taken as a whole, the data available for MIBC and metastatic UBC outcomes imply a pressing unmet need for improvements to what is currently achieved with systemic therapy.

Altered gene expression in cancer may arise through structural genomic changes, such as gene mutation or loss/gain of chromosomal content, or through reversible changes in the regulation and expression of genes. The latter, known as epigenetic control, includes biochemical modifications to the histone proteins adjacent to chromatin, and to DNA itself [9]. To date, DNA methylation at CpG di-nucleotides is the best studied epigenetic change in cancer. In general, there is a dysregulation of CpG methylation in cancerous cells that can lead to genomic instability and activation of previously silent oncogenes or silencing of tumour suppressor genes. In UBC, many genes are affected by promoter hypermethylation [10]. Reversal of this hypermethylation through DNA methyltransferase inhibition, or siRNA approaches, allows tumour suppressor gene re-expression and therefore holds promise as a potential anti-cancer therapy. Pre-clinical data from a variety of sources has shown that a strategy to combine a DNA demethylating agent with platinum-based chemotherapy might improve on current outcomes for UBC. These data indicate that, in addition to single agent activity, demethylating agents synergise with cisplatin and are able to circumvent cisplatin resistance in experimental models [11–16]. Data also exist to support investigation of a DNA hypomethylating agent with gemcitabine [17–21].

SGI-110 (guadecitabine, Astex Pharmaceuticals) is a DNA methyltransferase inhibitor composed of a dinucleotide of decitabine and deoxyguanosine formulated for subcutaneous injection. SGI-110 is in clinical development for a range of haematological and solid malignancies and is currently under investigation in phase I to III clinical trials. In a first in human phase I study, a maximum tolerated dose (MTD) was established at 90 mg/m^2^ daily × 5 in patients with myelodysplastic syndrome but was not reached in patients with acute myeloid leukaemia [22]. DNA demethylation was found to reach a plateau at 60 mg/m^2^ and this was designated as the biologically effective dose and recommended for phase II development. The most frequent grade 3 or higher adverse events were febrile neutropenia, pneumonia, thrombocytopenia, anaemia, and sepsis. Clinical responses to treatment were seen in some patients and confirmed in a subsequent phase II trial leading to an ongoing phase III study in acute myeloid leukaemia [23].

Decitabine and guadecitabine have been successfully combined with carboplatin with acceptable toxicity, epigenetic re-sensitisation and suggestion of a clinical benefit in heavily pre-treated ovarian cancer patients [24] (Matulonis UA, Oza A, Alvarez-Secord A, et al., unpublished observations). We hypothesise that cisplatin resistance might be reversed or avoided through co-administration with a DNA hypomethylating agent such as SGI-110.

## Methods/Design

The SPIRE trial is a phase Ib/IIa trial evaluating whether SGI-110 in combination with GC chemotherapy is safe and has a biologically effective dose.

### Objectives

The primary objective is to determine a recommended phase II dose (RP2D) for future phase II/III investigation as a neoadjuvant therapy in UBC. Furthermore, SPIRE aims to establish a RP2D for SGI-110 when combined with GC chemotherapy from both the MTD based on defined criteria for dose limiting toxicity (DLT) assessed by Common Terminology Criteria for Adverse Events (CTCAE) v4.03 and the maximally biologically effective dose (MBED) based on plasma/serum DNA LINE-1 methylation and haemoglobin F (HbF) re-expression status [25–27].

Secondary objectives include the evaluation of toxicity, assessed using CTCAE v4.03 at baseline, at each treatment cycle and at each follow-up visit; treatment compliance, assessed using electronic case report forms (eCRFs) during the treatment period; and pathological complete response rate (of bladder cancer patients enrolled in the phase IIa part of the trial).

Translational objectives include the evaluation of pharmacokinetic and pharmacodynamic effects of SGI-110 when combined with GC. Blood and bladder cancer tissue samples will be used to investigate (including but not limited to) promoter methylation of *MAGE-A1* and other cancer testis antigens and 5-methylcytosine levels and biomarkers of Nrf2 activation, including Nrf2, KEAP1 and Nrf2 transcriptional targets (e.g. glutathione reductase-1, metallothioneins, NQO-1).

### Study design

#### Phase Ib dose escalation component

**Fig. 1:**
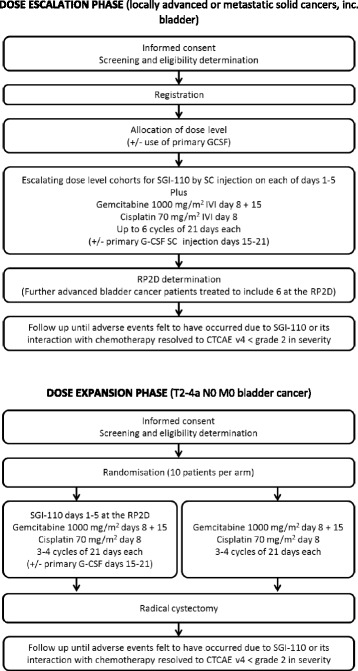
Trial schema

An initial dose escalation phase Ib component of the trial combines SGI-110 with GC chemotherapy in advanced solid tumours to establish a RP2D, using a modified rolling six-phase Ib design [28] (Fig. 1).

GC chemotherapy will be given for up to six cycles of a 21-day cycle. Cisplatin 70 mg/m^2^ will be administered on day 8 of each cycle by IV infusion over 2–4 h and gemcitabine 1000 mg/m^2^ on days 8 and 15 of each cycle by IV infusion over 30–60 min (and prior to cisplatin on day 8) (Table 1). Supportive treatment including anti-emetics and a suitable intravenous hydration schedule pre- and post-cisplatin will be administered according to local institutional policy.

**Table 1:** Treatment schedule over 21-day cycle

Day	1	2	3	4	5	6	7	8	9	10	11	12	13	14	15	16	17	18	19	20	21
SGI-110	X	X	X	X	X																
Cisplatin								X													
Gemcitabine								X							X						
(Granulocyte-colony stimulating factor)^a^															(X)	(X)	(X)	(X)	(X)	(X)	(X)

^a^Colony stimulating factor (G-CSF) administration: Patients in the initial dose level cohorts in the Dose Escalation Phase will not receive G-CSF as primary prophylaxis. If a patient in those initial cohorts is deemed to require G-CSF for a subsequent treatment cycle then they will discontinue SGI-110 and further use of cisplatin/gemcitabine will be at the discretion of the local investigator.Subsequent dose level cohorts may have G-CSF incorporated as primary prophylaxis for all patients according to the rules described under section 3.5. All subsequent patients will then have G-CSF (e.g. filgrastim or local equivalent) incorporated at a dose of 300 μg SC daily for 7 days from day 15 of each cycle

Four increasing dose levels of SGI-110 are planned (Table 2, dose level −1 is for those patients in dose level 1 requiring a dose reduction for treatment related toxicity). No intra-patient dose escalation will be undertaken.

**Table 2:** Dose cohort levels for the phase Ib dose escalation phase

Dose level	SGI-110 dose, per treatment cycle
−1	10 mg/m^2^, daily, on days 1–5
1	20 mg/m^2^, daily, on days 1–5
2	30 mg/m^2^, daily, on days 1–5
3	45 mg/m^2^, daily, on days 1–5
4	60 mg/m^2^, daily, on days 1–5

SGI-110 administration will be by sub-cutaneous injection, preferably in the abdominal area. Within each dose level cohort, 3–6 evaluable patients will be entered. A period of 21 days from the first patient starting treatment until treatment of subsequent patients will be observed.

Patients in initial dose level cohorts will not receive granulocyte-colony stimulating factor (G-CSF) as primary prophylaxis. If a DLT occurs, specifically due to neutropenia and/or its complications, then an additional 3–6 patients will be recruited to repeat the current dose level cohort with G-CSF incorporated as primary prophylaxis for all patients (e.g. filgrastim or local equivalent incorporated at a dose of 300 μg subcutaneous injection daily for 7 days from day 15 of each cycle) (Table 1). Subsequent cohorts will continue to use G-CSF as primary prophylaxis for all patients.

Dose modifications will be made for patients that do not meet certain haematological parameters on day of treatment together with dose modification criteria for non-haematological toxicities (Additional file 1 explains these dose modifications in more detail).

A DLT is assessed by CTCAE v4.03 and defined as any of the following events occurring between the first dose administration of SGI-110 and day 1 of the second cycle of treatment if related to the combination of SGI-110 and GC. The events are (1) greater than 14 days of delay in commencing a second cycle of treatment due to drug toxicity; (2) grade 4 neutropenia ≥ 7 days duration; (3) grade 3–4 neutropenia associated with a body temperature ≥ 38.5 °C; (4) grade 3–4 neutropenia associated with bacteriologically proven sepsis; (5) any grade 4 thrombocytopenia ≥ 7 days duration; (6) grade 3 thrombocytopenia associated with non-traumatic bleeding; or (7) any other clinically significant grade 3 or above toxicity except nausea or vomiting.

Criteria for dose escalation between patient cohorts, and determination of MTD, are defined in Table 3.

**Table 3:** Criteria for dose escalation between patient cohorts and determination of maximum tolerated dose (MTD)

Cohort size (evaluable patients)	Dose limiting toxicities in cycle 1	Actions
3–6	0	Cohorts 1–3: dose escalation to the next cohort Cohort 4: MTD is established at this dose level
< 6	1	Expand cohort to include up to 6 evaluable patients and re-evaluate
6	1	Cohorts 1–3: dose escalation to the next cohort Cohort 4: MTD is established at this dose
≥ 2	≥ 2	Dose level will be considered a non-tolerated dose; no further recruitment to this cohort and dose escalation will cease Cohort 1: The combination will be considered non-viable (consider incorporation of granulocyte-colony stimulating factor G-CSF) Cohorts 2–4: The previous dose level will be expanded to incorporate six evaluable patients (consider incorporation of G-CSF)

The MBED will be based on plasma/serum DNA *LINE-1* demethylation and HbF re-expression at day 8 of the chemotherapy cycle [22, 23, 29]. If a dose level is defined beyond which no further increase in demethylation of plasma/serum DNA *LINE-1* or re-expression of HbF is seen, then this will be defined as the MBED (if the MBED is above the MTD then no further dose escalation would occur and the MBED would remain undefined). Schedule may also be explored, in addition to dose, to optimise hypomethylation based on plasma/serum DNA *LINE-1* methylation and HbF re-expression status with respect to timing of cisplatin administration based on emergent data.

The RP2D will be established using the MTD and MBED according to the following principles: (1) if the MBED is clearly demonstrated to be below the MTD, then it will be utilised as the RP2D; (2) if the MBED and MTD are established to be equivalent, then this would be the RP2D; (3) if the MTD occurs at a dose at which at least a degree of plasma/serum DNA *LINE-1* demethylation and/or HbF re-expression occurs (but not necessarily maximal), then the MTD would be used as the RP2D; and (4) if the MTD occurs at a dose at which no plasma/serum DNA *LINE-1* demethylation or HbF re-expression is demonstrated, then this would result in discontinuation of development of this combination.

At least six evaluable patients are needed to determine RP2D. An evaluable patient is defined as one that, during cycle 1, has completed all relevant safety evaluation requirements and has received full doses of SGI-110 on days 1–5 and received full doses of GC on day 8 and has received at least one dose of G-CSF if the patient has been registered to a cohort with G-CSF prophylaxis in use. An evaluable patient is also a patient who has experienced a DLT. Once the RP2D has been determined, six additional patients will be recruited to assess the RP2D in patients with incurable advanced/metastatic UBC.

Depending on the tolerability of SGI-110 when combined with GC the number of patients recruited in the dose escalation phase Ib component of the trial will range from 3 to 36.

#### Phase IIa randomised component

If a suitable RP2D can be determined, the trial will proceed to an open label, randomised dose expansion phase IIa component in the neoadjuvant setting for UBC to evaluate either GC chemotherapy alone or in combination with the RP2D of SGI-110 identified in the dose escalation phase (Fig. 1). GC chemotherapy alone is considered routine standard treatment in the UK and, according to local practice, patients will receive up to 3 to 4 cycles of treatment. Patients will be randomised by clinic staff at site, 20 patients will be randomised using a web-based system in a 1:1 ratio using the method of permuted blocks. Treatment allocation will be unblinded. There will not be a formal statistical comparison between the two groups.

SPIRE will be run in three Experimental Cancer Medicine Centres in the UK with the aim of recruiting up to a total of 56 patients.

### Ethical and regulatory aspects

SPIRE has received ethical approval by the North West-Haydock Research Ethics Committee (15/NW/0936) and has approval from the UK Medicines and Health Care Product Regulatory Agency (MHRA). Southampton Clinical Trials Unit (SCTU), a Cancer Research UK core funded and UK Clinical Research Collaboration registered Clinical Trials Unit (CTU), is coordinating the trial. A list of recruiting sites can be obtained from the SCTU. University Hospital Southampton National Health Service Foundation Trust is the sponsor for the trial [30]. The SPIRE Trial Management Group (TMG) includes representatives from oncology, PPI representation and CTU staff involved in the day-to-day running of the trial. A SPIRE Safety Review Committee (SRC), comprising of oncology clinicians, statisticians and CTU staff, will review and assess safety, tolerability and any available pharmacokinetic and pharmacodynamic data to make protocol-defined decisions regarding trial progress during the dose escalation phase, including a decision to dose escalate or define the RP2D. The SRC will receive and review information on the progress and data relating to the trial and advise the TMG and Trial Steering Committee on the proposed conduct of the SPIRE trial dose escalation phase. In addition, an independent Trial Steering Committee is established, and a Data Monitoring and Ethics Committee comprising two clinicians and a statistician experienced in this research area will be constituted on the launch of the dose expansion phase to monitor trial progress and safety. Charters for these groups are available via spire@soton.ac.uk.

The SCTU has undertaken a risk assessment for the SPIRE trial which includes the requirements for monitoring (both central and site). The SCTU undertakes a number of internal audits of its own systems and processes annually and has routine audits from both its sponsor and the independent MHRA every 2–3 years.

The trial is registered on the UK NIHR trial portfolio, meaning there are research nurses based at UK cancer hospitals who help in screening potential patients to identify those eligible for the trial.

### Study participants

**Fig. 2:**
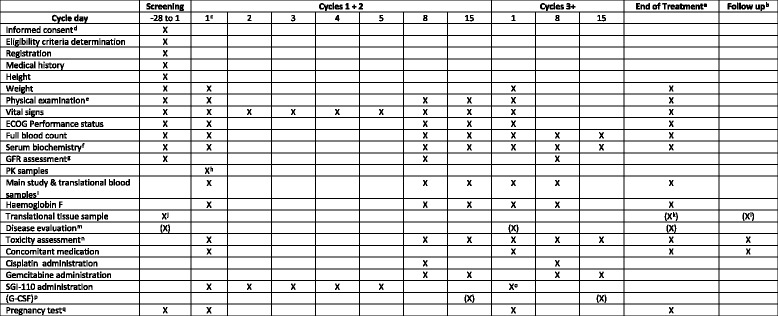
SPIRIT figure for SPIRE phase Ib dose escalation component

For the dose escalation phase I, the SPIRE trial is currently recruiting patients with incurable histologically or cytologically confirmed, locally advanced or metastatic, solid cancer, for which the use of GC is a clinically appropriate treatment in the view of the local principal investigator. Any number of previous lines of systemic chemotherapy is permitted. Within the dose expansion phase II component, we will recruit patients with bladder cancer with a pure or a predominant component of transitional cell carcinoma and clinical stage T2-4a N0 M0 who are planned to commence GC for 3 or 4 cycles with neoadjuvant (i.e. curative) intent prior to a planned radical cystectomy. No other investigational medicinal products should be received whilst on study and no live vaccines should be received during treatment and for 4 weeks following the end of treatment. In accordance with the cisplatin summary of product characteristics, nephrotoxic and ototoxic drugs are contraindicated during the course of the trial (see Fig. 2 and Table 4 for eligibility criteria).

**Table 4:** Eligibility criteria for the SPIRE trial

Inclusion criteria
All patients
1. Eastern Cooperative Oncology Group performance status of 0 or 1
2. Glomerular filtration rate estimation of ≥ 60 mL/min according to either the Cockcroft and Gault formula or by Cr-51 EDTA or Tc-99m DTPA clearance
3. Adequate haematological parameters:
• Haemoglobin ≥ 90 g/L
• Neutrophil count ≥ 1.5 × 10^9^/L
• Platelets ≥ 100 × 10^9^/L
4. Adequate biochemical parameters:
• Bilirubin ≤ 1.5 × upper limit of normal (ULN)
• ALT and ALP ≤ 2.5 × ULN (ALP ≤ 5 × ULN if caused by liver or bone metastases)
5. Aged 16 years or over
6. Life expectancy > 3 months
7. Provision of written informed consent
Patients in the dose escalation phase:
8. Incurable histologically or cytologically confirmed, locally advanced or metastatic, solid cancer, for which the use of gemcitabine and cisplatin is a clinically appropriate treatment in the view of the local principal investigator; any number of previous lines of systemic chemotherapy is permitted
Patients in the dose expansion phase:
9. Bladder cancer with a pure or predominant component of transitional cell carcinoma
10. Clinical stage T2-4a N0 M0
11. Planned to commence GC for 3 or 4 cycles with neoadjuvant (i.e. curative) intent prior to a planned radical cystectomy
Exclusion criteria
All patients
1. Unresolved toxicities from prior therapy greater than CTCAE v4.03 grade 1 (with the exception of alopecia) at the time of registration
2. Prior radiotherapy to > 30% of bone marrow
3. Major surgery within 30 days of registration/randomisation
4. Any investigational medicinal product within 30 days registration/randomisation
5. Allergy or other known intolerance to any of the proposed study drugs, including supportive agents and inclusive of G-CSF and locally utilised anti-emetics
6. Previously identified central nervous system metastases unless treated and clinically stable and not requiring steroids for at least 4 weeks prior to the start of trial treatment
7. Coronary artery bypass graft, angioplasty, vascular stent, myocardial infarction, unstable angina pectoris or congestive cardiac failure (New York Heart Association ≥ class II) within the last 6 months
8. Women who are pregnant or breast feeding (women of child-bearing potential must have a negative pregnancy test performed within 7 days prior to the start of trial treatment)
9. Patients of child-bearing potential who are not using a highly effective method of contraception
10. Any patient who, in the judgment of the local investigator, is unlikely to comply with trial procedures, restrictions or requirements
11. Any patient who has received a live vaccine within 4 weeks of initiation of their treatment
Patients in the dose expansion phase:
12. Recent or current separate other malignancy; current non-melanoma skin cancer, cervical carcinoma in situ or incidental localised prostate cancer is permissible; participants with a history of a separate other malignancy having completed all active treatment 2 or more years previously may be entered

#### Withdrawal criteria

Participants are free to withdraw consent from the study at any time without providing a reason. A participant could also withdraw from receiving study treatment but may not wish to withdraw from the trial. In this instance, the participant will be encouraged to attend follow-up visits in accordance with the trial schedule. Should any participant become pregnant during the trial, study treatment will be discontinued.

### Study procedure

#### Recruitment and consent

Patients are approached within a hospital setting and screened for eligibility by research staff to ensure all inclusion and exclusion criteria are met. Informed consent to enter the trial is obtained from a patient by a clinician only after a full explanation has been given, a patient information sheet provided and time allowed for consideration. Patients may provide written consent up to 42 days prior to registration. Patients are also asked to consent to provision of tumour and blood samples for use in laboratory studies, including genetic analysis, and for their data to be shared anonymously to support other research in the future (Additional files 2 and 3).

#### Baseline visit

**Fig. 3:**
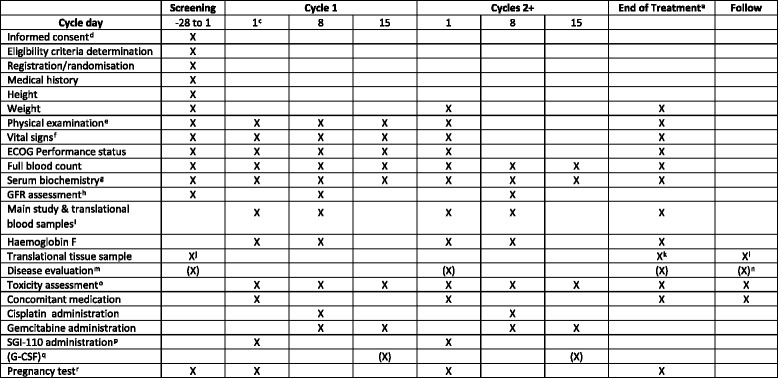
SPIRIT figure for SPIRE phase IIa dose expansion component (Additional file 4)

Following informed consent, assessments including a physical examination, full blood count, serum biochemistry, including renal, liver and bone profiles, and glomerular filtration rate are completed within 28 days prior to treatment commencing, with disease evaluation being undertaken in accordance with local policy and routine practice for the relevant disease site. Concomitant medications and medical history will be recorded. In addition, women of child-bearing potential will undertake a pregnancy test. Following registration/randomisation bloods and a formalin-fixed paraffin-embedded tumour tissue block will be obtained, either archival or fresh if no archival sample is available (see SPIRIT Figure, Figs. 2 and 3).

#### Treatment and follow-up visits

Participants attend hospital appointments for treatment cycles with assessments as performed in the baseline visit, plus adverse events, blood samples for translational analyses, and pharmacokinetics and treatment compliance. Following the treatment, phase participants enter a post-treatment/progression follow-up to collect data on adverse events, disease status and survival status. Serious adverse event reporting occurs in real-time to the SCTU safety desk throughout the study. Serious adverse events are assessed to determine whether they are related to drug treatment and unexpected or not, and subsequently reported to both Astex Pharmaceuticals and the UK regulatory bodies.

### Data collection

Research staff at hospitals complete trial eCRFs via a remote data collection tool (Medidata Rave). Data is checked for missing or unusual values and checked for consistency within participants over time by SCTU trial staff. Any suspect data are raised as data queries. Site staff respond to the queries providing an explanation/resolution to the discrepancies. Full details on data management procedures are available in the SPIRE Data Management Plan, available on request.

### Source document verification and monitoring

The trial will be monitored and audited in accordance with SCTU procedures. For the dose escalation phase, all patients registered will trigger a monitor from SCTU to visit site to source-verify data. This monitoring will encompass comparing entries on the trial eCRF with patients’ medical records and other supporting documents at site and documented in a monitoring report form. Details will remain confidential, consistent with data protection regulations. Drug accountability will also be monitored throughout the trial. For the dose expansion phase, the SCTU will use a risk-based monitoring process to determine monitoring frequency and extent.

### Statistical analysis

#### Dose escalation phase Ib

All patients entered into the phase Ib part of the trial will be accounted for. The phase Ib analysis will focus on the incidence of dose limiting toxicities, which will be summarised by dose cohort. In addition, worst recorded toxicity grade for each patient on the National Cancer Institute-CTCAE toxicity scale (version 4.03) during GC/SGI-110 treatment will be summarised by dose cohort. Details of dose delivery will also be summarised.

#### Dose expansion phase IIa

The analysis will be conducted in the intention-to-treat population, which includes all randomisation patients who have commenced study treatment. The analysis will not be powered for formal statistical comparisons of efficacy. Worst toxicity grade for each patient experienced during chemotherapy will be summarised by treatment arm and compared using the Mann–Whitney U test. Relative dose intensity of GC will be summarised by treatment arm. Pathological complete response rate according to local assessment will be summarised by treatment arm (the trial is not statistically powered for this). A formal statistical analysis plan will be developed prior to the end of the trial.

### Interim analysis

A SRC will review the data during the phase Ib component to determine dose escalation and RP2D. A Data Monitoring and Ethics Committee will monitor the trial during the phase IIa component where the safety, activity and treatment compliance analyses will be planned and agreed with the Data Monitoring and Ethics Committee in advance. It is anticipated that all patients who have been randomised will be included in these analyses.

All analyses will be carried out using STATA version 15 and SAS version 9 or later.

### Adverse event reporting

Data on adverse events are collected at treatment and follow-up visits. The trial also has a UK regulatory compliant real-time serious adverse events reporting process to identify serious adverse reactions and suspected unexpected serious adverse reactions that could suspend/stop the trial if warranted.

### End of the trial

The end of trial is defined as when the last patient has had their last data collected.

## Discussion

The outcome of this trial will provide evidence for whether SGI-110 in combination with GC chemotherapy is safe and a biologically effective dose to consider for future phase II/III trials in the neoadjuvant bladder cancer setting. The results may also be relevant for other cancers where GC chemotherapy is utilised. Results will be disseminated to patients and clinical teams through peer-reviewed journal publications authored by the member of the TMG and presented at international conferences.

### Trial status

This clinical trial was registered in December 2015 (EudraCT Number: 2015–004062-29, and in February 2016 on ISRCTN registry number: 16332228). Recruitment opened on May 5, 2016, and is expected to be completed in December 2018. The current protocol is version 3, dated 08-Dec-16. REC/MHRA-approved protocol amendments will be communicated to sites via email and updated trial documentation provided centrally via the trial website. Trial registries will be amended where relevant with explanations for these changes.

## Additional files

Additional file 1: Dose modification of SGI-110, gemcitabine and cisplatin guidance. This guidance is used if the patient does not meet certain haematological parameters or non-haematological toxicities have occurred. (PDF 107 kb)

Additional file 2: Copy of the consent form given to participants for phase I dose escalation. (PDF 161 kb)

Additional file 3: Copy of the consent form given to participants for phase II dose escalation. (PDF 161 kb)

Additional file 4: Standard Protocol Items: Recommendations for Interventional Trials (SPIRIT): a checklist for a set of scientific, ethical and administrative elements recommended to be listed in a protocol [31]. (PDF 58 kb)

## Abbreviations

CTCAE: Common Terminology Criteria for Adverse Events
CTU: Clinical Trials Unit
eCRF: electronic case report form
DLT: dose limiting toxicity
GC: gemcitabine/cisplatin
G-CSF: granulocyte-colony stimulating factor
HbF: haemoglobin F
MHRA: Medicines and Health Care Product Regulatory Agency
MIBC: muscle invasive bladder cancer
MBED: maximally biologically effective dose
MTD: maximum tolerated dose
RP2D: recommended phase II dose
SCTU: Southampton Clinical Trials Unit
SRC: Safety Review Committee
TMG: Trial Management Group
UBC: urothelial bladder cancer

## References

[1] Cancer Research UK. http://www.cancerresearchuk.org/health-professional/cancer-statistics/statistics-by-cancer-type/bladder-cancer. Accessed 26 Mar 2018.

[2] Grossman HB, Natale RB, Tangen CM, White RW, Sarosdy MF, Wood DP (2003). Neoadjuvant chemotherapy plus cystectomy compared with cystectomy alone for locally advanced. Bladder Cancer.

[3] Griffiths G, Hall R, Sylvester R, Raghavan D, Parmar MK, International Collaboration of Trialists, Medical Research Council Advanced Bladder Cancer Working Party (now the National Cancer Research Institute Bladder Cancer Clinical Studies Group), European Organisation for Research and Treatment of Cancer Genito-Urinary Tract Cancer Group, Australian Bladder Cancer Study Group, National Cancer Institute of Canada Clinical Trials Group, Finnbladder; Norwegian Bladder Cancer Study Group, Club Urologico Espanol de Tratamiento Oncologico Group (2011). International phase III trial assessing neoadjuvant cisplatin, methotrexate, and vinblastine chemotherapy for muscle-invasive bladder cancer: Long-term results of the BA06 30894 trial. J Clin Oncol.

[4] Von Der Maase H, Hansen SW, Roberts JT, Dogliotti L, Oliver T (2000). Gemcitabine and cisplatin versus methotrexate, vinblastine, doxorubicin, and cisplatin in advanced or metastatic bladder cancer: Results of a Large, randomized, multinational, multicenter, phase III study. J Clin Oncol.

[5] Crabb SJ, Wheater M (2010). Advances in chemotherapy and targeted systemic therapies for urothelial cancer. Curr Drug Ther.

[6] Vale CL (2005). Neoadjuvant chemotherapy in invasive bladder cancer: update of a systematic review and meta-analysis of individual patient data advanced bladder cancer (ABC) meta-analysis collaboration. Eur Urol.

[7] Von Der Maase H, Sengelov L, Roberts JT, Ricci S, Dogliotti L, Oliver T (2005). Long-term survival results of a randomized trial comparing gemcitabine plus cisplatin, with methotrexate, vinblastine, doxorubicin, plus cisplatin in patients with bladder cancer. J Clin Oncol.

[8] Bellmunt J, de Wit R, Vaughn DJ, Fradet Y, Lee JL, Fong L (2016). Pembrolizumab as second-line therapy for advanced urothelial carcinoma. N Engl J Med.

[9] Drayton RN, Dudziec E, Peter S, Hartmann A, Bryant HE, Catto JW (2014). Reduced expression of miRNA-27a modulates cisplatin resistance in bladder cancer by targeting the cystine/glutamate exchanger SLC7A11. Clin Cancer Res.

[10] Dudziec E, Gogl-Doring A, Cookson V, Chen W, Catto JWR (2012). Integrated epigenome profiling of repressive histone modifications DNA methylation and gene expression in normal and malignant urothelial cells. PLoS One.

[11] Cheng JC, Matsen CB, Gonzales FA, Ye W, Greer S, Marquez VE (2003). Inhibition of DNA methylation and reactivation of silenced genes by zebularine. J Natl Cancer Inst.

[12] Christoph F, Kempkensteffen C, Weikert S, Köllermann J, Krause H, Miller K (2006). Methylation of tumour suppressor genes APAF-1 and DAPK-1 and in vitro effects of demethylating agents in bladder and kidney cancer. Br J Cancer.

[13] Shang D, Liu Y, Matsui Y, Ito N, Nishiyama H, Kamoto T (2008). Demethylating agent 5-aza-2′-deoxycytidine enhances susceptibility of bladder transitional cell carcinoma to cisplatin. Urology.

[14] Ramachandran K, Gordian E, Singal R. 5-Azacytidine reverses drug resistance in bladder cancer cells. Anticancer Res. 2011;31:3757–66. 22110197

[15] Huihui Zhang, Fan Qi, Youhan Cao, Xiongbing Zu, Minfeng Chen, Zhuo Li and LQ. 5-Aza-2’Deoxycytidine Enhances Maspin Expression and Inhibits Proliferation, Migration, and Invasion of the Bladder Cancer T24 Cell Line. Cancer Biother Radiopharm. 2013;28:343–50. 10.1089/cbr.2012.1303 23570371

[16] Chang X, Monitto CL, Demokan S, Kim MS, Chang SS, Zhong X (2010). Identification of hypermethylated genes associated with cisplatin resistance in human cancers. Cancer Res.

[17] Tada Y, Yokomizo A, Shiota M, Tsunoda T, Plass C, Naito S (2011). Aberrant DNA methylation of T-cell leukemia, homeobox 3 modulates cisplatin sensitivity in bladder cancer. Int J Oncol.

[18] Samulitis BK, Pond KW, Pond E, Cress AE, Patel H, Wisner L (2015). Gemcitabine resistant pancreatic cancer cell lines acquire an invasive phenotype with collateral hypersensitivity to histone deacetylase inhibitors. Cancer Biol Ther.

[19] Füller M, Klein M, Schmidt E, Rohde C, Göllner S, Schulze I (2015). 5-azacytidine enhances efficacy of multiple chemotherapy drugs in AML and lung cancer with modulation of CpG methylation. Int J Oncol.

[20] Gray SG, Baird AM, O’Kelly F, Nikolaidis G, Almgren M, Meunier A (2012). Gemcitabine reactivates epigenetically silenced genes and functions as a DNA methyltransferase inhibitor. Int J Mol Med.

[21] Valdez BC, Nieto Y, Murray D, Li Y, Wang G, Champlin RE (2012). Epigenetic modifiers enhance the synergistic cytotoxicity of combined nucleoside analog-DNA alkylating agents in lymphoma cell lines. Exp Hematol.

[22] Issa J-PJ, Roboz G, Rizzieri D, Jabbour E, Stock W, O’Connell C (2015). Safety and tolerability of guadecitabine (SGI-110) in patients with myelodysplastic syndrome and acute myeloid leukaemia: a multicentre, randomised, dose-escalation phase 1 study. Lancet Oncol.

[23] Kantarjian HM, Roboz GJ, Kropf PL, Yee KWL, O’Connell CL, Tibes R (2017). Guadecitabine (SGI-110) in treatment-naive patients with acute myeloid leukaemia: phase 2 results from a multicentre, randomised, phase 1/2 trial. Lancet Oncol.

[24] Matei D, Fang F, Changyu S, Schilder J, Arnold A, Zeng Y (2012). Epigenetic resensitzation to platinum in ovarian cancer. Cancer Res.

[25] Aparicio A, North B, Barske L, Wang X, Bollati V, Weisenberger D (2009). LINE-1 methylation in plasma DNA as a biomarker of activity of DNA methylation inhibitors in patients with solid tumors. Epigenetics.

[26] Appleton K, Mackay HJ, Judson I, Plumb JA, McCormick C, Strathdee G (2007). Phase I and pharmacodynamic trial of the DNA methyltransferase inhibitor decitabine and carboplatin in solid tumors. J Clin Oncol.

[27] Sonpavde G, Aparicio AM, Zhan F, North B, DeLaune R, Garbo LE (2011). Azacitidine favorably modulates PSA kinetics correlating with plasma DNA LINE-1 hypomethylation in men with chemonaïve castration-resistant prostate cancer. Urol Oncol Semin Orig Investig.

[28] Skolnik JM, Barrett JS, Jayaraman B, Patel D, Adamson PC (2008). Shortening the timeline of pediatric phase I trials: the rolling six design. J Clin Oncol.

[29] Clouser CL, Holtz CM, Mullett M, Crankshaw DL, Briggs JE, O’Sullivan MG (2012). Activity of a novel combined antiretroviral therapy of gemcitabine and decitabine in a mouse model for HIV-1. Antimicrob Agents Chemother.

[30] University Hospital Southampton website. http://www.uhs.nhs.uk/Research/Research.aspx. Accessed 18 Jan 2018.

[31] Chan A-W, Tetzlaff JM, Altman DG, Laupacis A, Gøtzsche PC, Krleža-Jerić K (2013). SPIRIT 2013 Statement: Defining standard protocol items for clinical trials. Ann Intern Med.

