# Supplementary material for: SPIRE – combining SGI-110 with cisplatin and gemcitabine chemotherapy for solid malignancies including bladder cancer: study protocol for a phase Ib/randomised IIa open label clinical trial
Source: Trials. 2018 Apr 3;19:216. doi: 10.1186/s13063-018-2586-7 (PMC5883402; doi:10.1186/s13063-018-2586-7)
Supplement: Supplementary file 1 — Dose modification of SGI-110, gemcitabine and cisplatin guidance. This guidance is used if the patient does not meet certain haematological parameters or non-haematological toxicities have occurred. (PDF 107 kb) [file 13063_2018_2586_MOESM1_ESM.pdf]

DOSE MODIFICATIONS FOR HAEMATOLOGICAL TOXICITY

Modifications of drug timings and doses will be made as follows. Once dose reductions have been made then doses will not be re-escalated during subsequent cycles.

Criteria For Treatment To Occur On Day 1 and 8

At the point of treatment on day 1 (i.e. for administration of SGI-110 for days 1-5) and on day 8 (cisplatin and gemcitabine) of each cycle, haematological and non–haematological toxicities must have resolved to within the following limits for further treatment to occur. If these parameters are not met then treatment will be deferred up to a maximum of 14 days. During such a period of deferred treatment the patient should undergo weekly review (or more frequent if clinically appropriate) and the relevant parameter, and any others that are deemed important, should be reassessed. If a delay of > 14 days occurs then SGI-110 will be discontinued permanently and further use of cisplatin/gemcitabine will at the discretion of the local investigator:

- absolute neutrophil count ≥ 1.0 x 10^9/L;
- and platelet count ≥ 100 x 10^9/L;
- and non-haematological toxicities have resolved to ≤ grade 2

Criteria For Treatment To Occur On Day 15

Treatment on day 15 (gemcitabine alone) will be according to the following table

Neutrophils (x 10^9/L)		Platelets (x 10^9/L)	Gemcitabine
≥ 1.0	and	≥ 75	Continue current existing dose
0.5 to <1.0	and/or	50-74	Reduce by 25% the dose on this and future cycles
<0.5	or	<50	Omit on this cycle

Haematological Criteria For Subsequent Dose Reductions

If during the preceding treatment cycle any of the following occur then SGI-110 should be reduced by one dose level and gemcitabine should be reduced by 25% on day 8 and day 15 for all subsequent cycles:

- Grade 3 – 4 neutropenia associated with a temperature ≥ 38.5°C or bacteriologically proven sepsis
- Grade 3 thrombocytopenia associated with non-traumatic bleeding
- Any grade 4 thrombocytopenia

If a patient meets the criteria above on a subsequent cycle (and after the relevant dose reduction) or requires a greater than 14 day delay in starting a treatment cycle then SGI-110 will be discontinued permanently and further use of cisplatin/gemcitabine will at the discretion of the local investigator.

DOSE MODIFICATIONS FOR NON-HAEMATOLOGICAL TOXICITY

Neurotoxicity: is a recognised complication of cisplatin. Dose modifications for cisplatin should not be routinely made on the basis of grade 1 or 2 neurotoxicity. If participants experience grade 3 or 4 neurotoxicity then protocol treatment, including SGI-110, should be permanently stopped. Further off-trial chemotherapy will be at the discretion of the local investigator.

Renal toxicity: is a recognised complication of cisplatin. Serum creatinine should be measured prior to cisplatin on cycle 2 onwards and used to calculate GFR (ml/min) according to the Cockcroft and Gault formula. Dose adjustments for renal function should be made according to the following table.

Calculated GFR	Dose (cycles 2 onwards)
≥ 60ml/min<br>and<br>≥ 80% of base-line (pre-cycle 1)	Full dose
< 60ml/min<br><br>or<br><br>< 80% of base-line value	Consider hydration, avoidance of other nephrotoxic drugs and correct if possible.<br><br>Continue treatment only if an uncorrected isotopic GFR is > 60ml/min. If GFR remains <60ml/min, the participant should discontinue trial therapy. Further off-trial chemotherapy will be at the discretion of the local investigator

Liver toxicity: Liver transaminitis (elevated alanine or aspartate transaminase) may occur following gemcitabine chemotherapy. Day 8 treatment will be delayed and day 15 gemcitabine doses will be omitted in case of grade 3 or 4 transaminitis (i.e. ALT or AST > 5 x upper limit of normal). Further gemcitabine should not be given until toxicity has resolved to grade 2 or less and gemcitabine doses should be reduced by 25% with subsequent cycles of treatment. If a greater than 14 day delay in day 8 treatment occurs then trial treatment will be discontinued permanently and further use of cisplatin/gemcitabine will at the discretion of the local investigator. Careful monitoring of liver function should be performed if alanine or aspartate transaminase become elevated during treatment.

Other non-haematological toxicities not previously specified:

Grade 1 or 2:

- Begin appropriate symptomatic care, e.g. anti-emetics, anti-diarrhoeals, laxatives, emollient creams, etc. as clinically indicated
- Delay of up to 14 days may be undertaken at the discretion of the local investigator to allow reduction of toxicity to ≤ grade 1 severity
- Maintain dose level

Grade 3 or 4:

With the exception of any non-haematological toxicity previously specified, treatment dose modifications should be made according to the following criteria.

- Begin appropriate symptomatic care
- Interrupt trial treatment until toxicity is resolved to grade 1 or less

- Decrease dose of SGI-110 by one dose level with subsequent cycles. Reduction of cisplatin and gemcitabine or both may also be reduced (by 25% each) at the discretion of the local investigator and with respect to the perceived likely causative agent.

If any grade non-haematological toxicity has not resolved to grade 1 or less within 14 days, then treatment within the SPIRE trial should be discontinued. Further treatment off-trial will remain at the discretion of the responsible clinician.
