# Supplementary material for: SPIRE – combining SGI-110 with cisplatin and gemcitabine chemotherapy for solid malignancies including bladder cancer: study protocol for a phase Ib/randomised IIa open label clinical trial
Source: Trials. 2018 Apr 3;19:216. doi: 10.1186/s13063-018-2586-7 (PMC5883402; doi:10.1186/s13063-018-2586-7)
Supplement: Supplementary file 3 — Copy of the consent form given to participants for phase II dose escalation. (PDF 161 kb) [file 13063_2018_2586_MOESM3_ESM.pdf]

CANCER RESEARCH UK

<TO BE PRINTED ON LOCAL HOSPITAL HEADED PAPER>

SPIRE

INFORMED CONSENT FORM - PHASE II

Patient ID Number:
(to be obtained post registration) ______________________________

Name of Researcher:

____

Title of Project: SGI-110 to potentiate platinum response: A phase Ib/randomised IIa open label clinical trial combining SGI-110 with cisplatin and gemcitabine chemotherapy for solid malignancies including bladder cancer

Please *initial* each box

1. I confirm that I have read and understand the patient information sheet dated **XX – Month - Year,** version _**V XXX**_ _ _for the above study and I fully understand what is involved in taking part in this trial. I have had the opportunity to ask questions and these have been answered satisfactorily ☐

2. I understand that my participation is voluntary and that I am free to withdraw at any time, without giving a reason and without my medical care or legal rights being affected. ☐

3. I understand that relevant sections of my medical records, and data collected during the study, may be looked at by individuals from the Sponsor or their delegates, from regulatory authorities, from Astex Pharmaceuticals Inc. (the USA-based company who manufacture and supply SGI-110) or from the NHS Trust where it is relevant to my taking part in this research. I give permission for these individuals to have access to my records. ☐

4. I understand that the information collected about me will be used to support other research in the future, and may be shared anonymously with other researchers. ☐

5. I consent to my anonymised trial data being used in future research by third parties involved in this research, including those both inside and outside the European Economic Area (for example the USA). ☐

6. I agree to my General Practitioner being informed of my participation in the study. ☐

7. I agree to give extra samples of blood for the main study, as described in the patient information sheet. I give permission for my samples, and other information with my details, to be transferred to relevant organisations for analysis (these may be outside of the European Economic Area).

8. I agree to use effective contraception as detailed in the patient information sheet for the above study.

9. I understand that I shall not benefit financially in any way by taking part in this study.

10. I agree to my pseudo-anonymised data being held on servers located in the EU and USA. Access to this data will be strictly controlled by Southampton Clinical Trials Unit (SCTU) and applicable Data Protection Legislation will be abided by.

11. I agree to take part in the above study.

WOMEN:

Please initial each box:

12. I understand that I will need to take a pregnancy test at screening as detailed in the patient information sheet for the above study.

OPTIONAL:

Please initial each box:

13. I understand that the information held and maintained by NHS Digital (formally known as Health and Social Care Information Centre) and other central UK NHS bodies may be used to help contact me or provide information about my health status. I give permission for this information to be obtained and stored by Southampton Clinical Trials Unit to enable long term follow-up.

14. I give my permission for an archived tissue sample from my previous biopsy/surgery for cancer to be collected for research purposes, as described in the information sheet for the above study. I give permission for these samples, a copy of my consent form and other information with my details to be transferred to the University of Southampton Cancer Sciences Tissue Bank.

15. I give my permission for tissue samples collected as part of the routine care/surgery for my cancer, to be collected for research purposes, as described in the information sheet for the above study. I give permission for these samples, a copy of my consent form and other information with my details to be transferred to the University of Southampton Cancer Sciences Tissue Bank.

16. I agree that samples of my blood can be used for translational research purposes as described in the patient information sheet for the above study. I understand that giving samples is voluntary and that I am free to withdraw my consent for use of the samples at any time without giving a reason and without my medical care or legal rights being affected. ☐

17. I agree that the blood samples, tissue samples and information collected about me will be stored on behalf of the SPIRE Trial Management Group and may be used in future ethically approved projects. I understand that some of these projects may be carried out by researchers other than the SPIRE Trial Management Group. ☐

------------------------------	------------------------------	------------------------------
Name of Patient	Signature	Date

------------------------------	------------------------------	------------------------------
Name of Person taking consent	Signature	Date

REMINDER FOR RESEARCH TEAM:

- **Original signed consent form in Investigator Site File**
- **One copy given to the patient**
- **One copy filed in the patient's medical records**
